# Geographic patterns in patient demographics and insulin use in 18 countries, a global perspective from the multinational observational study assessing insulin use: understanding the challenges associated with progression of therapy (MOSAIc)

**DOI:** 10.1186/s12902-015-0044-z

**Published:** 2015-09-09

**Authors:** Jennifer M. Polinski, Seoyoung C. Kim, Dingfeng Jiang, Ahmed Hassoun, William H. Shrank, Xavier Cos, Efraín Rodríguez-Vigil, Shuichi Suzuki, Ikuro Matsuba, John D. Seeger, Wesley Eddings, Gregory Brill, Bradley H. Curtis

**Affiliations:** CVS Health, Woonsocket, RI USA; Division of Pharmacoepidemiology and Pharmacoeconomics, Department of Medicine, Brigham and Women’s Hospital, and Harvard Medical School, 1620 Tremont St, Suite 3030, Boston, MA 02120 USA; Harvard Medical School, Boston, MA USA; Eli Lilly and Company, Indianapolis, IN USA; Dubai Diabetes Center, Dubai, UAE; CAP Sant Marti de Provencals, Catalan Institute of Health, Barcelona, Spain; Center for Diabetes Control, Inc, Carolina, PR ᅟ; Eli Lilly Japan K.K., Kobe, Japan; Kanagawa Physicians Association, Kanagawa, Japan; Harvard School of Public Health, Boston, MA USA

## Abstract

**Background:**

Among patients with type 2 diabetes, insulin intensification to achieve glycemic targets occurs less often than clinically indicated. Barriers to intensification are not well understood. We present patients’ baseline characteristics from MOSAIc, a study investigating patient-, physician-, and healthcare environment-based factors affecting insulin intensification and subsequent health outcomes.

**Methods:**

MOSAIc is a longitudinal, observational study following patients’ diabetes care in 18 countries: United Arab Emirates (UAE), Argentina, Brazil, Canada, China, Germany, India, Israel, Italy, Japan, Mexico, Russia, Saudi Arabia, South Korea, Spain, Turkey, United Kingdom, United States. Eligible patients are age ≥18, have type 2 diabetes, and have used insulin for ≥3 months with/without other antidiabetic medications. Extensive baseline demographic, clinical, and psychosocial data are collected at baseline and regular intervals during the 24-month follow-up. We conducted descriptive analyses of baseline data.

**Results:**

Four thousand three hundred forty one patients met eligibility criteria. Patients received their type 2 diabetes diagnosis 12 ± 8 years prior to baseline visit, yet patients in developing countries were younger than in developed countries (e.g., UAE, 55 ± 10; Germany = 70 ± 10). Saudi Arabians had the highest HbA1c values (9.0 ± 2.2) and Germany (7.5 ± 1.4) among the lowest. Most patients in 5 (28 %) of the 18 countries did not use an oral antidiabetic drug. Over half of patients in fourteen (78 %) countries exclusively used basal insulin; most Indian and Chinese patients exclusively used mixed insulin.

**Conclusions:**

MOSAIc’s baseline data highlight differences in patient characteristics across countries. These patterns, along with physician and healthcare environment differences, may contribute to the likelihood of insulin intensification and subsequent clinical outcomes.

**Electronic supplementary material:**

The online version of this article (doi:10.1186/s12902-015-0044-z) contains supplementary material, which is available to authorized users.

## Background

Both the global incidence and prevalence of type 2 diabetes are increasing rapidly, and cases are expected to reach 329 million by 2030 [1]. The most striking increases have been in developing countries. In Saudi Arabia today, 24 % of citizens have type 2 diabetes, a 10-fold increase in prevalence since 1982 [2, 3]. Current type 2 diabetes prevalence exceeds 8 % in Brazil, China, India, and the Russian Federation, and exceeds 12 % in Mexico, Turkey, and the United Arab Emirates [4]. The morbidity and mortality burden as well as the economic toll associated with type 2 diabetes are substantial. Type 2 diabetes is a major risk factor for heart disease and stroke and the fifth leading cause of death worldwide [5–7]. In the U.S. alone, type 2 diabetes-related health expenditures exceeded $174 billion in 2007, with 1/3 of those expenditures, $58 billion, spent on preventable complications [8].

Anti-hyperglycemic medications can effectively reduce blood glucose levels and prevent or forestall diabetes-related complications, yet poor disease control is common, especially when patients’ progressive disease merits the addition of insulin therapy [9–12]. Among the barriers to insulin initiation and glycemic control are patients’ injection fears and perceived social stigma; providers’ concerns about hypoglycemia, weight gain, and patients’ ability to follow more complex regimens; and health system factors, such as patients’ out-of-pocket costs for and access to medications [12–17]. Preliminary cross-sectional evidence suggests that barriers to and facilitators for insulin intensification, e.g., adding bolus doses and/or increasing injection frequency, are distinct from those for insulin initiation. At present, there is no longitudinal evidence to identify and quantify barriers to insulin intensification. Because many patients’ glycemic levels merit such intensification to achieve glycemic targets over time, these barriers must be enumerated and addressed [5, 18–20].

The 2-year, longitudinal MOSAIc (Multinational Observational Study Assessing Insulin use: understanding the challenges associated with progression of therapy) study was designed to identify patient, physician, and health system factors that influence insulin intensification among patients with type 2 diabetes and to quantify the relationships between these factors and long-term clinical outcomes [21]. In this paper, we describe and explore the heterogeneity of baseline demographic, clinical, and psychosocial characteristics among 4,341 MOSAIc patients enrolled in 18 different countries. These data provide a unique, holistic glimpse into the clinical and psychosocial experience of patients with type 2 diabetes worldwide, and lay the foundation for MOSAIc’s longitudinal analyses.

## Research design and methods

### Study design

The design of, study population, and data collection for the MOSAIc study have been described extensively [21]. In brief, MOSAIc is a 2-year, longitudinal observational study that follows patients’ real world diabetes care and health outcomes. MOSAIc involves no additional treatments, visits, or laboratory collections beyond those occurring within the course of normal care. Data collection occurs during an initial baseline visit and during four subsequent prospective visit windows (within ±3 months) at 6, 12, 18, and 24 months. At present, only baseline data are available for all patients.

### Study population

**Fig. 1:**
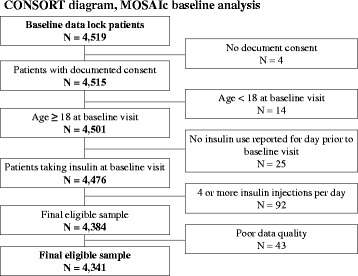
CONSORT diagram for MOSAIc baseline analytic population

MOSAIc has enrolled 4,519 patients from primary care or specialty practice sites across 18 countries [United Arab Emirates (UAE), Argentina, Brazil, Canada, China, Germany, India, Israel, Italy, Japan, Mexico, Russia, Saudi Arabia, South Korea, Spain, Turkey, United Kingdom (UK), United States including Puerto Rico (US)]. These participating countries were chosen based on geographic region, population aged 20–79, and the T2DM prevalence in each region and country [21]. These countries have heterogeneous levels of economic development, industrialization, and healthcare accessibility [22] and represent 5 global regions: Asia (China, India, Japan, South Korea), Europe (Germany, Italy, Russia, Spain, UK), North America (Canada, US), Middle East/North Africa (Israel, Saudi Arabia, Turkey, UAE), South/Central America (Argentina, Brazil, Mexico). Patients were recruited from two types of clinics, primary care practices and diabetes specialty clinics, to be representative of each participating country’s diabetic patient population. In addition, we included sites in both rural and urban locations and academic and non-academic settings. Clinicians treating enrolled patients have also contributed demographic data and patient-specific treatment goals at baseline. MOSAIc-eligible patients were 1) age **≥** 18; 2) taking any commercially-available insulin therapy other than intensive basal-bolus insulin therapy (i.e., basal + 3 prandial injections) from any manufacturer for **≥** 3 months with or without any combination of approved non-insulin antidiabetic medications; 3) were not simultaneously participating in a study that includes an investigational drug or procedure; and 4) were proficient in the country’s primary language [21]. All enrolled patients gave documented consent. Due to inconsistent and poor data quality, 2 practice sites have been closed since the study began. Data from these sites were excluded from analyses in the present manuscript (Fig. 1). Institutional Review Board approvals were obtained in all countries [21]. The Institutional Review Board of the Brigham and Women’s Hospital, the data coordinating center for the entire MOSAIc study, deemed the study’s analytic plan as exempt from review. A list of the ethics review boards for all countries is provided in Additional file 1.

### Baseline data collection

Each patient’s available type 2 diabetes clinical history, including diagnosis date, treatment and complications, and medication history, was assessed retrospectively from the practice site’s medical record. Type 2 diabetes-related health care resource utilization (physician visits, hospitalizations, and auxiliary provider visits: diabetes educators, ophthalmologists, podiatrists, cardiologists, dietitians, and nephrologists), most recent recorded laboratory, biometric, and vital sign values, and other comorbidities were also assessed, but limited to the period 6 months prior to the baseline visit. Patients’ type 2 diabetes medication regimen at the time of the baseline visit, including medication name, dose, frequency of use, method of administration (oral, syringe, pen, or pump) were collected. Finally, each patient’s physician reported an HbA1c goal for him/her.

Extensive information on patients’ diabetes—and insulin-related knowledge, attitudes, and behaviors; hypoglycemia and fasting; general health behaviors; patient-provider relationships; and perceived physical and psychological well being were collected at baseline using self-report questionnaires. In this paper, we focus on 3 of these validated surveys. The Diabetes Knowledge Test examines patients’ understanding of their disease, such as how to manage insulin use and how to treat hypoglycemia, with a summary score ranging from 0 (no questions correct) to 9 (all correct) [23]. The 17-item Diabetes Distress Scale asks patients to indicate to what degree aspects of their type 2 diabetes treatment and care are of concern, using a 6-point Likert scale ranging from “Not a problem” to “A very serious problem [24].” The summary score ranges from 17 (no distress) to 102 (severe distress). The 25-item Interpersonal Processes of Care survey measures patients’ perceived quality of their relationship with their providers; scores range from 1 (poor relationship) to 5 (good relationship) [25]. We also examined a single question regarding self-monitoring of blood glucose from the Summary of Diabetes Self-Care Activities questionnaire: “On how many of the last seven days did you test your blood sugar the number of times recommended by your health-care provider?”; responses ranged from 0 to 7 [26].

### Statistical analyses

From the collected data, we created several additional variables of interest. The first was the summed injection frequency across insulin medications; if insulin injection frequency was missing, a once daily frequency was assumed. If method of insulin administration was missing, we assumed that a syringe was used. We classified patients as using a syringe, pen, or pump for insulin administration; syringe was reported if multiple methods were used (i.e., syringe and pen or syringe and pump). Only 12 patients were using an insulin pump alone; these patients’ insulin administration type was excluded from the present analysis.

We performed descriptive analyses (mean ± standard deviation for continuous data; number and percentage for categorical data) for baseline variables of interest, for all patients and then by individual country. Due to the observational nature of the MOSAIc study, baseline data were not available for all patients for all variables of interest. The median proportion of missing data for any one variable was 4.9 %, interquartile range, 3.6–7.8 %. Patterns of missingness appeared to vary by country. Based on these facts, we used two approaches in our analyses. In the first, a “complete case” analysis, we calculated the descriptive measure among those patients for whom a value was present for a given variable of interest. Our second approach used multiple imputation via chained equations to accommodate continuous as well as categorical variables [27]. Ten imputed datasets were created. The multiple imputation models included all variables of interest for which complete data were available as well as indicator variables for each country to account for country-specific variation. Imputation was done for the variable with the smallest proportion of missingness first and then in order of increasing missingness for other variables with missing data. To report descriptive statistics using the imputation approach, categorical and continuous values were converted to the log scale to approximate a normal distribution, averaged across the 10 imputed datasets, and then converted back to the linear scale; therefore, values and proportions for each level of the categorical values may not sum to the total number of patients or to 100 %. All analyses were performed using SAS 9.3 (SAS Institute; Cary, NC) and Stata 13 (StataCorp LP; College Station, TX).

## Results

Among 4,519 patients with baseline data, 4,341 met all MOSAIc eligibility criteria and comprised the analytic population (Fig. 1). The most common exclusion was patients’ use of four or more insulin injections per day, *N =* 92 (2 %). Because results using the complete case analytic approach were similar to those using multiple imputation, and space constraints preclude presenting results for all 18 countries, our manuscript tables present multiple imputation results for 7 countries that represent developed and developing economies, large patient populations, and varied races, ethnicities, and geographic regions (China, India, Mexico, Germany, the Russian Federation, Saudi Arabia, and the US). Multiple imputation results for the remaining 11 countries are presented in supplementary tables found in Online Supplement 2; results from the complete case approach are also available in the online supplement.

Patients’ demographic characteristics varied significantly across countries (Table 1, Additional file 2: Table S1, S2). UAE (mean age, 55 ± 10) and Saudi Arabian (57 ± 10) patients were among the youngest, while German patients were, on average, 15 years older. 70 % of Russian patients were female versus only 39 % of Japanese patients. Few or no patients reported that they were uninsured in Germany (0 %), the US (5 %), and Israel (7 %), but 62 % of Italian patients reported having no insurance. Current alcohol use was modest in 17 countries: 5 % (India) – 31 % (Canada); but in the UK, 51 % reported current use. Current smokers made up 6 % of Indian versus 22 % of Turkish patients. On average, MOSAIc patients received their type 2 diabetes diagnosis 12 ± 8 years prior to their baseline visit (Table 2, Additional file 2: Table S3, S4). In 12 of 18 countries, patients’ mean BMI (kg/m^2^) was ≥30, indicating obesity; in the remaining 6 countries, patients’ mean BMI was between 25 and 29 [28]. Clinically meaningful differences in baseline HbA1c levels (%) were observed: on average, patients in Saudi Arabia had the highest HbA1c values (9.0 ± 2.2) followed closely by Turkey (8.9 ± 2.0); Germany (7.5 ± 1.4) and China (7.6 ± 1.8) had the lowest. Even though most countries’ patients had poor glycemic control, there was little variability in their healthcare providers’ HbA1c goals for them, mean 6.9 ± 0.6. There were remarkable differences in the prevalence of concurrent diagnoses as reported in the medical record: only 2 % of Mexican patients had CAD or CHF, but 55 % of Russian patients had CAD and 34 % had CHF. Neuropathy was present in at least two-fifths of American, South Korean, Russian, and Saudi Arabian patients, but more rarely noted in Argentina (9 %) and Spain (12 %).

**Table 1:** Baseline demographic characteristics of 4,341 patients enrolled in the MOSAIc study

	All patients *N =* 4,341	China *N =* 373	Germany *N =* 149	India *N =* 918	Mexico *N =* 206	Russia *N =* 240	Saudi Arabia *N =* 226	USA *N =* 540
	*N (%) or mean ± SD*							
Demographics								
Age, years	61 ± 11	60 ± 10	70 ± 10	58 ± 10	60 ± 12	64 ± 10	57 ± 10	64 ± 12
Female gender	2176 (50)	209 (56)	69 (46)	405 (44)	117 (57)	168 (70)	135 (60)	284 (53)
Education								
<12 grade	1982 (46)	186 (50)	115 (77)	373 (41)	122 (59)	32 (13)	99 (44)	122 (23)
High school graduate or partial college	1327 (31)	132 (35)	20 (13)	203 (22)	41 (20)	92 (38)	56 (25)	270 (50)
≥ College degree	1032 (24)	55 (15)	15 (10)	342 (37)	43 (21)	116 (49)	71 (31)	148 (27)
Marital status								
Married or living with a significant other	3490 (80)	342 (92)	107 (72)	860 (94)	154 (75)	167 (69)	177 (78)	355 (66)
Single	851 (20)	31 (8)	42 (28)	58 (6)	52 (25)	73 (31)	49 (22)	185 (34)
Employment								
Manual labor	1119 (26)	60 (16)	18 (12)	222 (24)	86 (42)	19 (8)	85 (37)	87 (16)
Professional labor	1358 (31)	161 (43)	23 (16)	284 (31)	59 (29)	135 (56)	83 (37)	178 (33)
Skilled labor	1865 (43)	152 (41)	108 (72)	411 (45)	61 (29)	86 (36)	59 (26)	276 (51)
Insurance type								
Private	1010 (23)	38 (10)	1 (1)	299 (33)	33 (16)	6 (3)	2 (1)	343 (63)
Public	2424 (56)	297 (80)	148 (99)	160 (17)	117 (57)	221 (92)	167 (74)	171 (32)
Uninsured	907 (21)	38 (10)	0 (0)	459 (50)	56 (27)	13 (5)	57 (25)	27 (5)
Alcohol consumption								
Current	619 (14)	39 (11)	31 (21)	47 (5)	30 (14)	3 (1)	1 (0)	151 (28)
Past	1044 (24)	92 (25)	40 (27)	93 (10)	45 (22)	15 (6)	1 (0)	244 (45)
Smoking status								
Current	587 (14)	67 (18)	17 (11)	55 (6)	33 (16)	24 (10)	15 (7)	92 (17)

**Table 2:** Baseline clinical characteristics of 4,341 patients enrolled in MOSAIc study, as recorded in the patient medical record

	All patients *N =* 4,341	China *N =* 373	Germany *N =* 149	India *N =* 918	Mexico *N =* 206	Russia *N =* 240	Saudi Arabia *N =* 226	USA *N =* 540
	*N (%) or mean ± SD*							
Diabetes duration, in years	12 ± 8	11 ± 7	14 ± 8	12 ± 8	13 ± 9	10 ± 7	11 ± 7	13 ± 8
*Physician’s* HbA1c goal for the patient (%)	6.9 ± 0.6	6.6 ± 0.9	6.9 ± 0.5	6.9 ± 0.5	6.7 ± 0.7	6.7 ± 0.5	7.2 ± 0.4	6.8 ± 0.5
*Physician’s* HbA1c goal for the patient (mmol/mol)	52 ± 7	49 ± 10	52 ± 6	52 ± 6	50 ± 8	50 ± 6	55 ± 4	51 ± 6
Laboratory values								
HbA1c level (%)	8.2 ± 1.8	7.6 ± 1.8	7.5 ± 1.4	8.6 ± 1.7	8.6 ± 2.2	7.7 ± 1.3	9.0 ± 2.2	8.0 ± 1.6
HbA1c level (mmol/mol)	66 ± 20	60 ± 20	58 ± 15	70 ± 19	70 ± 24	61 ± 14	75 ± 24	64 ± 18
Biometric measurements								
Systolic blood pressure (mmHg)	132.4 ± 16.2	130.2 ± 15.7	137.9 ± 17.1	131.2 ± 14.5	130.5 ± 17.5	136.3 ± 11.9	134.5 ± 13.3	132.0 ± 18.4
Body mass index (kg/m^2^)	30 ± 6	25 ± 3	30 ± 5	27 ± 5	28 ± 5	32 ± 5	32 ± 6	34 ± 8
Diabetes-related complications								
Amputation	50 (1)	0 (0)	3 (2)	4 (0)	4 (2)	3 (1)	2 (1)	10 (2)
Gastroparesis	111 (3)	11 (3)	2 (1)	7 (1)	3 (2)	18 (8)	24 (11)	13 (2)
Nephropathy	720 (17)	70 (19)	37 (25)	75 (8)	17 (8)	60 (25)	51 (23)	105 (19)
Neuropathy	1280 (29)	118 (32)	57 (38)	224 (24)	50 (24)	155 (65)	90 (40)	216 (40)
Retinopathy	1026 (24)	85 (23)	22 (15)	143 (16)	32 (15)	117 (49)	120 (53)	79 (15)
Diagnostic history of:								
Coronary artery disease	808 (19)	75 (20)	30 (20)	105 (11)	3 (2)	132 (55)	59 (26)	140 (26)
Congestive heart failure	244 (6)	21 (6)	19 (13)	19 (2)	3 (1)	81 (34)	5 (2)	34 (6)
Depression	401 (9)	13 (4)	13 (9)	22 (2)	27 (13)	16 (7)	9 (4)	122 (23)
Hypertension	3045 (70)	199 (53)	134 (90)	630 (69)	114 (55)	208 (87)	143 (63)	465 (86)
Hyperlipidemia	2562 (59)	204 (55)	97 (65)	392 (43)	89 (43)	144 (60)	155 (69)	444 (82)
Myocardial infarction	284 (7)	7 (2)	10 (7)	21 (2)	5 (3)	47 (20)	18 (8)	46 (8)
Stroke	157 (4)	18 (5)	10 (7)	11 (1)	1 (0)	14 (6)	4 (2)	32 (6)

More than half of patients in China, Mexico, Germany, Russia and Turkey did not use an oral antidiabetic drug (Table 3, Additional file 2: Table S5, S6). While more than two-thirds of patients in Japan, Mexico, Spain, Italy, Brazil, and Russia exclusively used basal insulin, more than half of Indian and Chinese patients exclusively used mixed insulin. Across countries, once daily insulin injection was most commonly observed; however, in China and India, twice daily insulin injection was most the most frequent regimen.

**Table 3:** Characteristics of insulin and oral antidiabetic medication use among patients, by country

	All patients *N =* 4,341	China *N =* 373	Germany*N =* 149	India *N =* 918	Mexico *N =* 206	Russia *N =* 240	Saudi Arabia *N =* 226	USA *N =* 540
	*N (%)*							
Any insulin regimen together with:								
0 oral antidiabetic drugs	1449 (33)	203 (54)	90 (60)	161 (18)	120 (58)	127 (53)	56 (25)	149 (28)
1 oral antidiabetic drug	1388 (32)	109 (29)	41 (28)	243 (27)	58 (28)	69 (29)	41 (18)	224 (41)
2 oral antidiabetic drugs	977 (23)	52 (14)	14 (9)	282 (31)	21 (10)	37 (15)	78 (35)	132 (24)
3 or more oral antidiabetic drugs	527 (12)	9 (2)	4 (3)	232 (25)	7 (3)	7 (3)	51 (23)	35 (6)
Insulin regimen								
Basal insulin only	2234 (51)	60 (16)	65 (44)	252 (27)	146 (71)	197 (82)	140 (62)	355 (66)
Basal + short-acting insulin only	237 (5)	3 (1)	6 (4)	11 (1)	15 (7)	13 (5)	1 (0)	32 (6)
Mixed insulin only	1310 (30)	249 (67)	66 (44)	522 (57)	34 (17)	19 (8)	40 (18)	93 (17)
Short-acting insulin only	175 (4)	13 (4)	5 (3)	43 (5)	9 (4)	10 (4)	0 (0)	20 (4)
Other insulin combinations	385 (9)	48 (13)	7 (5)	90 (10)	2 (1)	1 (0)	45 (20)	40 (7)
Insulin injection frequency								
Once per day	2579 (59)	128 (34)	80 (54)	415 (45)	152 (74)	161 (67)	147 (65)	373 (69)
Twice per day	1585 (37)	220 (59)	58 (39)	494 (54)	51 (25)	77 (32)	78 (35)	144 (27)
Three times per day	177 (4)	25 (7)	11 (7)	9 (1)	3 (2)	2 (1)	1 (0)	23 (4)
Insulin delivery device								
Pen	3220 (74)	372 (100)	141 (95)	541 (59)	67 (33)	220 (93)	142 (63)	243 (45)
Syringe	1109 (26)	1 (0)	8 (5)	373 (41)	138 (68)	17 (7)	84 (37)	296 (55)

On average, patients’ scores on the Diabetes Knowledge Test ranged from 3 ± 2 questions (comparatively low knowledge) correct in South Korea to 7 ± 2 questions correct in China; scores range from 1 (little or no knowledge) to 9 (very good knowledge) (Table 4, Additional file 2: Table S7, S8). Patients’ Diabetes Distress Scale scores ranged from a mean 24 ± 9 (minimal distress) in Germany to 55 ± 21 (moderate distress) in Turkey; patients in Brazil (54 ± 22), Saudi Arabia (51 ± 17) and UAE reported similar moderate distress. Reported self-monitoring of blood glucose at least once a day over 7 days ranged between 1 ± 2 days in India to 6 ± 2 in Canada. In contrast to the variation observed for these 2 measures, patients uniformly reported an ambivalent relationship with their healthcare providers; the mean score on the Interpersonal Processes of Care survey in all 18 countries was 3.

**Table 4:** Self-reported outcomes among patients, by country

	All patients *N =* 4,341	China *N =* 373	Germany *N =* 149	India *N =* 918	Mexico *N =* 206	Russia *N =* 240	Saudi Arabia *N =* 226	USA *N =* 540
	*mean ± SD*							
Diabetes Knowledge Test score^a^	5 ± 2	7 ± 2	6 ± 2	4 ± 2	4 ± 2	6 ± 2	4 ± 2	5 ± 2
Diabetes Distress Scale score^b^	38 ± 19	28 ± 11	24 ± 9	37 ± 19	39 ± 21	49 ± 18	51 ± 17	33 ± 15
Interpersonal Processes of Care score^c^	3 ± 0.5	3 ± 0.5	3 ± 0.5	3 ± 0.5	3 ± 0.6	3 ± 0.5	3 ± 0.5	3 ± 0.4
Self-monitoring of blood glucose^d^	3 ± 3	2 ± 2	5 ± 2	1 ± 2	3 ± 3	4 ± 2	2 ± 2	5 ± 3

^**a**^ The Diabetes Knowledge Test’s summary score ranges from 0 (no questions correct) to 9 (all questions correct)

^**b**^ The Diabetes Distress Scale score ranges from 17 (no distress) to 102 (severe distress)

^**c**^ The Interpersonal Processes of Care score ranges from 1 (poor relationship with healthcare provider) to 5 (good relationship with healthcare provider)

^**d**^ The self monitoring of blood glucose value ranges from 0 (checked blood glucose on no days of the week as recommended by healthcare provider) to 7 (checked blood glucose on all 7 days of the week as recommended by healthcare provider)

## Discussion

Among 4,341 patients with type 2 diabetes taking insulin who are participating in the MOSAIc observational study, we found substantial variation across patients’ baseline demographic characteristics, medical history, drug treatment regimens, and self-reported knowledge of and distress about diabetes. Acknowledging these patient-specific differences, our aggregate results also showed striking country-specific differences in patients’ profiles and treatment patterns, offering insight into the healthcare environments in each geographic setting. These data also highlight the complex interplay of demographic, medical and self-reported data with health status and type 2 diabetes treatment and underscore the limitations of a one-size-fits-all approach to improving type 2 diabetes management, insulin adherence, and clinical outcomes.

Age, gender, and disease history are among the strongest predictors of adverse health outcomes. While across countries, MOSAIc patients had been diagnosed with type 2 diabetes on average 12 years before study enrollment, potentially because all were using insulin at enrollment, it is important to highlight that patients in developing countries like Brazil, UAE, Saudi Arabia, and Mexico were still significantly younger at enrollment than were patients in more developed nations such as Germany. Type 2 diabetes is a progressive disease, and as such, an increasingly intensive treatment strategy is needed to achieve glycemic control over time, with likely insulin intensification. Because of its prospective, longitudinal design, MOSAIc is well positioned to inform these treatment progressions and to assess which treatment progression patterns lead to better clinical outcomes than others. This type of evidence will be of use when clinical treatment guidelines are updated.

In addition to demographic differences across countries, we observed significant variation in the report of diagnoses in patients’ medical records as well as patients’ self-reported type 2 diabetes knowledge and distress. Variation in the prevalence of reported medical diagnoses may be attributable to differences across countries in the completeness of medical records, providers’ medical reporting practices, and/or true absence of disease in the MOSAIc patient population. Because MOSAIc is an observational study, these potential explanations cannot be disentangled. Variations in patients’ self-report data may reflect differences in education, the social acceptability of acknowledging distress, and the availability of blood glucose test strips with which to self-monitor glucose levels. These variations are rooted not only in patients’ heterogeneity, but also in the heterogeneity of the larger healthcare environment. The documentation of these differences among patients and across geographic locations and their relative contributions to type 2 diabetes outcomes and disease progression may draw healthcare providers and health policymakers’ attention to their importance and the need to more rigorously record them.

We observed the most striking variation across type 2 diabetes treatment regimens. Oral antidiabetic medication use, insulin type and injection frequency, and insulin delivery mode all differed by country. While patients’ type 2 diabetes profile certainly plays a role in treatment planning, it is likely that differences in countries’ healthcare environments account for an important proportion of the variation we observed. Market penetration of certain medications versus others, the pricing of medications, the proportion of patients with insurance, and the availability of syringes versus pens with which to inject insulin, and the availability of glucose test strips to determine blood glucose levels may all play a role. Our planned longitudinal analyses will investigate the contribution of these and other healthcare environment factors on patients’ treatment regimens and regimen intensification over time [21]. These analyses and the role of specific treatment patterns in patients’ adherence to their type 2 diabetes medication regimens will also inform improved educational materials, insulin use training guides, and care and storage of insulin medications.

The study has several limitations. As an observational study with a wide breadth and depth of data collection from both medical records and via self-report, baseline data were not available for all patients for all variables of interest. We used multiple imputation with chained equations, a well-recognized imputation method, which accommodates both categorical and continuous variables, to impute missing values. This approach assumes that the missing values are missing at random, but it is not possible to test this assumption. For these reason, we also used a complete case analysis approach. Results using the multiple imputation approach and results using a complete case approach were quantitatively similar. While enrolled patients’ demographic, clinical, and psychosocial characteristics may be different from those of patients with type 2 diabetes in the general population of each country [21], we recruited patients from both endocrinology and primary care practice sites with varied practice locations (urban/rural), size, and practice types (academic/stand-alone) to maximize generalizability. MOSAIc study is an observational cohort in which physicians provide usual care to their patients, reflecting characteristics and patterns of type 2 diabetes patients and their treatments in real-world settings. In addition, unlike randomized controlled trials of therapeutic agents, MOSAIc’s exclusion criteria are not extensive [21]. Ongoing analyses will compare MOSAIc results to results in the general population in countries where they are available and to results in other published studies. These comparisons are outside the scope of the present manuscript.

## Conclusion

MOSAIc is the first global study of its kind to collect and integrate patient-, provider- and healthcare environment-level factors that may influence patients’ type 2 diabetes treatment regimens and disease course. The baseline patient characteristics presented here reflect the underlying heterogeneity across these levels at MOSAIc enrollment and hint at the complexity of optimizing patients’ type 2 diabetes disease management and health outcomes. Longitudinal data collection and analysis during the 2 years of the MOSAIc study will help untangle this complexity and contribute to our understanding and treatment of type 2 diabetes.

## Additional files

Additional file 1:
**Names of Ethics Review Boards granting approval to MOSAIc, by country.** (DOCX 23 kb) Additional file 2: (DOCX 87 kb)
