# Supplementary material for: Geographic patterns in patient demographics and insulin use in 18 countries, a global perspective from the multinational observational study assessing insulin use: understanding the challenges associated with progression of therapy (MOSAIc)
Source: BMC Endocr Disord. 2015 Sep 9;15:46. doi: 10.1186/s12902-015-0044-z (PMC4563845; doi:10.1186/s12902-015-0044-z)
Supplement: Additional file 2: — (DOCX 87 kb) [file 12902_2015_44_MOESM2_ESM.docx]

This is supplement is divided into two parts: Multiple Imputed Tables and Complete Case Tables. The first four tables of the Multiple Imputed Tables are provided in text while the remaining eight are only provided here. The last twelve tables are the Complete Case Tables that are only provided here.

**Multiple Imputed Tables**

**Supplementary Table 1:** Baseline demographic characteristics of 4,341 patients enrolled in the MOSAIc study

|  | **All patients**  **N=4341** | **Argentina**  **N=160** | **Brazil**  **N=155** | **Canada**  **N=149** | **Israel**  **N=100** | **Italy**  **N=160** |
| --- | --- | --- | --- | --- | --- | --- |
|  | *N (%) or mean ± SD* | | | | | |
| **Demographics** |  |  |  |  |  |  |
| Age, years | 61 ± 11 | 65 ± 11 | 61 ± 10 | 66 ± 11 | 62 ± 9 | 64 ± 11 |
| Female gender | 2176 (50) | 76 (48) | 100 (65) | 63 (42) | 45 (45) | 60 (38) |
| Education |  |  |  |  |  |  |
| <12 grade | 1982 (46) | 101 (63) | 91 (59) | 67 (45) | 32 (32) | 104 (65) |
| High school graduate or partial college | 1327 (31) | 34 (21) | 33 (21) | 66 (45) | 48 (48) | 49 (31) |
| > College degree | 1032 (24) | 25 (16) | 31 (20) | 16 (11) | 20 (20) | 8 (5) |
| Marital status |  |  |  |  |  |  |
| Married or living with a significant other | 3490 (80) | 115 (72) | 95 (61) | 105 (70) | 87 (87) | 128 (80) |
| Single | 851 (20) | 45 (28) | 60 (39) | 44 (30) | 13 (13) | 32 (20) |
| Employment |  |  |  |  |  |  |
| Manual labor | 1119 (26) | 56 (35) | 66 (42) | 45 (30) | 19 (19) | 39 (24) |
| Professional labor | 1358 (31) | 34 (21) | 55 (36) | 39 (26) | 37 (37) | 33 (21) |
| Skilled labor | 1865 (43) | 70 (44) | 34 (22) | 65 (44) | 44 (44) | 88 (55) |
| Insurance type |  |  |  |  |  |  |
| Private | 1010 (23) | 57 (36) | 54 (35) | 45 (30) | 19 (19) | 8 (5) |
| Public | 2424 (56) | 67 (42) | 83 (53) | 77 (51) | 74 (74) | 53 (33) |
| Uninsured | 907 (21) | 36 (22) | 18 (12) | 27 (18) | 7 (7) | 99 (62) |
| Alcohol consumption |  |  |  |  |  |  |
| Current | 619 (14) | 29 (18) | 15 (10) | 46 (31) | 1 (1) | 31 (19) |
| Past | 1044 (24) | 38 (24) | 28 (18) | 76 (51) | 1 (1) | 58 (36) |
| Smoking status |  |  |  |  |  |  |
| Current | 587 (14) | 17 (11) | 14 (9) | 20 (13) | 13 (13) | 34 (21) |

**Supplementary Table 2:** Baseline demographic characteristics of 4,341 patients enrolled in the MOSAIc study

|  | **All patients**  **N=4341** | **Japan**  **N=157** | **South Korea**  **N=141** | **Spain**  **N=148** | **Turkey**  **N=283** | **UAE**  **N=110** | **UK**  **N=126** |
| --- | --- | --- | --- | --- | --- | --- | --- |
|  | *N (%) or mean ± SD* | | | | | | |
| **Demographics** |  |  |  |  |  |  |  |
| Age, years | 61 ± 11 | 64 ± 13 | 62 ± 11 | 68 ± 12 | 58 ± 9 | 55 ± 10 | 65 ± 11 |
| Female gender | 2176 (50) | 61 (39) | 51 (36) | 78 (53) | 142 (50) | 54 (49) | 59 (47) |
| Education |  |  |  |  |  |  |  |
| <12 grade | 1982 (46) | 36 (23) | 66 (47) | 121 (81) | 205 (73) | 40 (36) | 72 (57) |
| High school graduate or partial college | 1327 (31) | 90 (57) | 53 (38) | 22 (15) | 55 (19) | 29 (27) | 35 (28) |
| > College degree | 1032 (24) | 31 (20) | 22 (16) | 5 (4) | 23 (8) | 41 (37) | 20 (15) |
| Marital status |  |  |  |  |  |  |  |
| Married or living with a significant other | 3490 (80) | 119 (76) | 124 (88) | 112 (75) | 249 (88) | 97 (88) | 99 (79) |
| Single | 851 (20) | 38 (24) | 17 (12) | 36 (25) | 34 (12) | 13 (12) | 27 (21) |
| Employment |  |  |  |  |  |  |  |
| Manual labor | 1119 (26) | 25 (16) | 56 (40) | 64 (43) | 128 (45) | 18 (16) | 27 (21) |
| Professional labor | 1358 (31) | 45 (29) | 27 (19) | 16 (11) | 49 (17) | 65 (59) | 36 (28) |
| Skilled labor | 1865 (43) | 86 (55) | 58 (41) | 69 (46) | 107 (38) | 28 (25) | 63 (50) |
| Insurance type |  |  |  |  |  |  |  |
| Private | 1010 (23) | 43 (27) | 13 (9) | 1 (1) | 2 (1) | 33 (30) | 11 (9) |
| Public | 2424 (56) | 101 (64) | 115 (82) | 145 (98) | 279 (98) | 70 (64) | 82 (65) |
| Uninsured | 907 (21) | 14 (9) | 13 (9) | 2 (1) | 2 (1) | 7 (6) | 34 (27) |
| Alcohol consumption |  |  |  |  |  |  |  |
| Current | 619 (14) | 62 (39) | 34 (24) | 20 (14) | 9 (3) | 6 (5) | 64 (51) |
| Past | 1044 (24) | 101 (64) | 59 (42) | 41 (28) | 18 (6) | 8 (7) | 88 (70) |
| Smoking status |  |  |  |  |  |  |  |
| Current | 587 (14) | 40 (26) | 27 (19) | 21 (14) | 63 (22) | 16 (15) | 19 (15) |

**Supplementary Table 3:** Baseline clinical characteristics of 4,341 patients enrolled in MOSAIc study, as recorded in the patient medical record

|  | **All patients**  **N=4341** | **Argentina**  **N=160** | **Brazil**  **N=155** | **Canada**  **N=149** | **Israel**  **N=100** | **Italy**  **N=160** |
| --- | --- | --- | --- | --- | --- | --- |
|  | *N (%) or mean ± SD* | | | | | |
| Diabetes duration, in years | 12 ± 8 | 13 ± 10 | 13 ± 8 | 15 ± 8 | 14 ± 8 | 13 ± 8 |
| *Physician’s* HbA1c goal for the patient (%, mmHg) | 6.9 ± 0.6 | 7.0 ± 0.5 | 7.0 ± 0.4 | 6.9 ± 0.5 | 7.2 ± 0.6 | 6.7 ± 0.6 |
| *Physician’s* HbA1c goal for the patient (mmol/mol) | 52 ± 7 | 53 ± 6 | 53 ± 4 | 52 ± 6 | 55 ± 7 | 50 ± 7 |
|  |  |  |  |  |  |  |
| **Laboratory values** |  |  |  |  |  |  |
| HbA1c level (%, mmHg) | 8.2 ± 1.8 | 8.1 ± 1.7 | 8.2 ± 1.7 | 7.9 ± 1.3 | 7.9 ± 1.5 | 8.0 ± 1.4 |
| HbA1c level (mmol/mol) | 66 ± 20 | 65 ± 19 | 66 ± 19 | 63 ± 14 | 63 ± 16 | 64 ± 15 |
|  |  |  |  |  |  |  |
| **Biometric measurements** |  |  |  |  |  |  |
| Systolic blood pressure (mmHg) | 132.4 ± 16.2 | 133.8 ± 13.1 | 135.7 ± 18.5 | 132.0 ± 16.5 | 129.6 ± 15.1 | 135.3 ± 17.9 |
| Body mass index (kg/m^2^) | 30 ± 6 | 31 ± 6 | 30 ± 5 | 32 ± 7 | 30 ± 6 | 29 ± 5 |
|  |  |  |  |  |  |  |
| **Diabetes-related complications** |  |  |  |  |  |  |
| Amputation | 50 (1) | 4 (3) | 7 (4) | 0 (0) | 2 (2) | 1 (1) |
| Gastroparesis | 111 (3) | 4 (2) | 3 (2) | 4 (3) | 0 (0) | 1 (1) |
| Nephropathy | 720 (17) | 14 (9) | 19 (12) | 29 (20) | 26 (26) | 23 (14) |
| Neuropathy | 1280 (29) | 14 (9) | 24 (16) | 38 (25) | 18 (18) | 37 (23) |
| Retinopathy | 1026 (24) | 24 (15) | 26 (17) | 32 (21) | 27 (27) | 54 (34) |
|  |  |  |  |  |  |  |
| **Diagnostic history of:** |  |  |  |  |  |  |
| Coronary artery disease | 808 (19) | 14 (9) | 13 (9) | 38 (25) | 28 (28) | 29 (18) |
| Congestive heart failure | 244 (6) | 0 (0) | 3 (2) | 4 (3) | 8 (8) | 4 (3) |
| Depression | 401 (9) | 4 (3) | 22 (14) | 24 (16) | 7 (7) | 9 (6) |
| Hypertension | 3045 (70) | 112 (70) | 113 (73) | 126 (84) | 76 (76) | 113 (71) |
| Hyperlipidemia | 2562 (59) | 81 (51) | 97 (62) | 113 (76) | 87 (87) | 97 (61) |
| Myocardial infarction | 284 (7) | 12 (8) | 7 (4) | 19 (13) | 19 (19) | 15 (10) |
| Stroke | 157 (4) | 4 (3) | 6 (4) | 6 (4) | 10 (10) | 8 (5) |

**Supplementary Table 4:** Baseline clinical characteristics of 4,341 patients enrolled in MOSAIc study, as recorded in the patient medical record

|  | **All patients**  **N=4341** | **Japan**  **N=157** | **South Korea**  **N=141** | **Spain**  **N=148** | **Turkey**  **N=283** | **UAE**  **N=110** | **UK**  **N=126** |
| --- | --- | --- | --- | --- | --- | --- | --- |
|  | *N (%) or mean ± SD* | | | | | | |
| Diabetes duration, in years | 12 ± 8 | 14 ± 9 | 15 ± 9 | 13 ± 8 | 11 ± 7 | 15 ± 8 | 12 ± 6 |
| *Physician’s* HbA1c goal for the patient (%, mmHg) | 6.9 ± 0.6 | 6.3 ± 0.6 | 7.1 ± 0.6 | 7.0 ± 1.0 | 6.8 ± 0.6 | 6.9 ± 0.2 | 7.4 ± 0.4 |
| *Physician’s* HbA1c goal for the patient (mmol/mol) | 52 ± 7 | 45 ± 7 | 54 ± 7 | 53 ± 11 | 51 ± 7 | 52 ± 2 | 57 ± 4 |
|  |  |  |  |  |  |  |  |
| **Laboratory values** |  |  |  |  |  |  |  |
| HbA1c level (%, mmHg) | 8.2 ± 1.8 | 7.7 ± 1.3 | 8.0 ± 1.3 | 8.0 ± 1.5 | 8.9 ± 2.0 | 8.2 ± 1.7 | 8.4 ± 1.7 |
| HbA1c level (mmol/mol) | 66 ± 20 | 61 ± 14 | 64 ± 14 | 64 ± 16 | 74 ± 22 | 66 ± 19 | 68 ± 19 |
|  |  |  |  |  |  |  |  |
| **Biometric measurements** |  |  |  |  |  |  |  |
| Systolic blood pressure (mmHg) | 132.4 ± 16.2 | 131.3 ± 15.5 | 126.6 ± 15.5 | 134.9 ± 16.4 | 131.5 ± 17.6 | 131.4 ± 17.4 | 136.1 ± 18.9 |
| Body mass index (kg/m^2^) | 30 ± 6 | 25 ± 4 | 26 ± 4 | 30 ± 5 | 31 ± 5 | 32 ± 7 | 33 ± 6 |
|  |  |  |  |  |  |  |  |
| **Diabetes-related complications** |  |  |  |  |  |  |  |
| Amputation | 50 (1) | 0 (0) | 1 (1) | 1 (1) | 5 (2) | 0 (0) | 2 (2) |
| Gastroparesis | 111 (3) | 5 (3) | 0 (0) | 9 (6) | 4 (1) | 1 (1) | 2 (2) |
| Nephropathy | 720 (17) | 44 (28) | 50 (36) | 29 (20) | 31 (11) | 24 (22) | 16 (13) |
| Neuropathy | 1280 (29) | 25 (16) | 61 (43) | 18 (12) | 71 (25) | 43 (39) | 23 (18) |
| Retinopathy | 1026 (24) | 40 (26) | 70 (50) | 29 (20) | 45 (16) | 33 (30) | 48 (38) |
|  |  |  |  |  |  |  |  |
| **Diagnostic history of:** |  |  |  |  |  |  |  |
| Coronary artery disease | 808 (19) | 13 (8) | 22 (16) | 23 (16) | 40 (14) | 18 (17) | 27 (21) |
| Congestive heart failure | 244 (6) | 4 (3) | 1 (1) | 25 (17) | 3 (1) | 1 (1) | 8 (7) |
| Depression | 401 (9) | 5 (3) | 13 (9) | 25 (17) | 22 (8) | 13 (12) | 34 (27) |
| Hypertension | 3045 (70) | 87 (55) | 97 (69) | 112 (76) | 123 (43) | 90 (82) | 103 (82) |
| Hyperlipidemia | 2562 (59) | 95 (61) | 89 (63) | 97 (66) | 104 (37) | 100 (91) | 78 (62) |
| Myocardial infarction | 284 (7) | 13 (8) | 9 (6) | 9 (6) | 11 (4) | 6 (5) | 9 (7) |
| Stroke | 157 (4) | 5 (3) | 9 (6) | 5 (3) | 2 (1) | 6 (5) | 5 (4) |

**Supplementary Table 5:** Characteristics of insulin and oral antidiabetic medication use among patients, by country

|  | **All patients**  **N=4341** | **Argentina**  **N=160** | **Brazil**  **N=155** | **Canada**  **N=149** | **Israel**  **N=100** | **Italy**  **N=160** |
| --- | --- | --- | --- | --- | --- | --- |
|  | *N (%)* | | | | | |
| **Any insulin regimen together with:** |  |  |  |  |  |  |
| 0 oral antidiabetic drugs | 1449 (33) | 72 (45) | 32 (21) | 55 (37) | 25 (25) | 33 (21) |
| 1 oral antidiabetic drug | 1388 (32) | 61 (38) | 59 (38) | 42 (28) | 41 (41) | 46 (29) |
| 2 oral antidiabetic drugs | 977 (23) | 25 (16) | 55 (36) | 31 (21) | 23 (23) | 41 (26) |
| 3 or more oral antidiabetic drugs | 527 (12) | 2 (1) | 9 (6) | 21 (14) | 11 (11) | 40 (25) |
| **Insulin regimen** |  |  |  |  |  |  |
| Basal insulin only | 2234 (51) | 103 (64) | 122 (79) | 92 (62) | 65 (65) | 118 (74) |
| Basal + short-acting insulin only | 237 (5) | 9 (6) | 30 (19) | 8 (5) | 16 (16) | 17 (11) |
| Mixed insulin only | 1310 (30) | 36 (23) | 0 (0) | 24 (16) | 6 (6) | 3 (2) |
| Short-acting insulin only | 175 (4) | 1 (1) | 1 (1) | 0 (0) | 0 (0) | 6 (4) |
| Other insulin combinations | 385 (9) | 11 (7) | 2 (1) | 25 (17) | 13 (13) | 16 (10) |
| **Insulin injection frequency** |  |  |  |  |  |  |
| Once per day | 2579 (59) | 71 (44) | 79 (51) | 84 (56) | 73 (73) | 123 (77) |
| Twice per day | 1585 (37) | 68 (43) | 56 (36) | 55 (37) | 20 (20) | 20 (13) |
| Three times per day | 177 (4) | 21 (13) | 20 (13) | 10 (7) | 7 (7) | 17 (11) |
| **Insulin delivery device** |  |  |  |  |  |  |
| Pen | 3220 (74) | 159 (99) | 26 (17) | 144 (97) | 83 (80) | 137 (85) |
| Syringe | 1109 (26) | 1 (1) | 129 (83) | 5 (3) | 17 (17) | 24 (15) |

**Supplementary Table 6:** Characteristics of insulin and oral antidiabetic medication use among patients, by country

|  | **All patients**  **N=4341** | **Japan**  **N=157** | **South Korea**  **N=141** | **Spain**  **N=148** | **Turkey**  **N=283** | **UAE**  **N=110** | **UK**  **N=126** |
| --- | --- | --- | --- | --- | --- | --- | --- |
|  | *N (%)* | | | | | | |
| **Any insulin regimen together with:** |  |  |  |  |  |  |  |
| 0 oral antidiabetic drugs | 1449 (33) | 29 (19) | 50 (36) | 39 (26) | 168 (59) | 13 (12) | 27 (21) |
| 1 oral antidiabetic drug | 1388 (32) | 35 (22) | 70 (50) | 75 (51) | 82 (29) | 25 (23) | 67 (53) |
| 2 oral antidiabetic drugs | 977 (23) | 34 (22) | 19 (14) | 27 (18) | 31 (11) | 48 (44) | 27 (21) |
| 3 or more oral antidiabetic drugs | 527 (12) | 59 (38) | 2 (1) | 7 (5) | 2 (1) | 24 (22) | 5 (4) |
| **Insulin regimen** |  |  |  |  |  |  |  |
| Basal insulin only | 2234 (51) | 110 (70) | 81 (57) | 106 (72) | 85 (30) | 66 (60) | 71 (56) |
| Basal + short-acting insulin only | 237 (5) | 11 (7) | 12 (9) | 18 (12) | 11 (4) | 23 (21) | 1 (1) |
| Mixed insulin only | 1310 (30) | 17 (11) | 14 (10) | 9 (6) | 123 (43) | 10 (9) | 45 (36) |
| Short-acting insulin only | 175 (4) | 13 (8) | 4 (3) | 9 (6) | 36 (13) | 1 (1) | 4 (3) |
| Other insulin combinations | 385 (9) | 6 (4) | 30 (21) | 6 (4) | 28 (10) | 10 (9) | 5 (4) |
| **Insulin injection frequency** |  |  |  |  |  |  |  |
| Once per day | 2579 (59) | 114 (73) | 107 (76) | 91 (62) | 208 (74) | 80 (73) | 93 (74) |
| Twice per day | 1585 (37) | 37 (24) | 33 (23) | 54 (37) | 71 (25) | 20 (18) | 29 (23) |
| Three times per day | 177 (4) | 6 (4) | 1 (1) | 3 (2) | 4 (1) | 10 (9) | 4 (3) |
| **Insulin delivery device** |  |  |  |  |  |  |  |
| Pen | 3220 (74) | 157 (100) | 134 (95) | 147 (99) | 282 (100) | 108 (98) | 122 (98) |
| Syringe | 1109 (26) | 0 (0) | 7 (5) | 1 (1) | 1 (0) | 2 (2) | 4 (3) |

**Supplementary Table 7:** Self-reported outcomes among patients, by country

|  | **All patients**  **N=4341** | **Argentina**  **N=160** | **Brazil**  **N=155** | **Canada**  **N=149** | **Israel**  **N=100** | **Italy**  **N=160** |
| --- | --- | --- | --- | --- | --- | --- |
|  | *mean ± SD* | | | | | |
| **Diabetes Knowledge Test score^*^** | 5 ± 2 | 5 ± 2 | 4 ± 2 | 5 ± 2 | 5 ± 2 | 6 ± 2 |
|  |  |  |  |  |  |  |
| **Diabetes Distress Scale score^†^** | 38 ± 19 | 36 ± 20 | 54 ± 22 | 27 ± 12 | 42 ± 19 | 37 ± 17 |
|  |  |  |  |  |  |  |
| **Interpersonal Processes of Care score^‡^** | 3 ± 0.5 | 3 ± 0.4 | 3 ± 0.5 | 3 ± 0.4 | 3 ± 0.5 | 3 ± 0.4 |
|  |  |  |  |  |  |  |
| **Self-monitoring of blood glucose^§^** | 3 ± 3 | 5 ± 3 | 3 ± 3 | 6 ± 2 | 4 ± 3 | 5 ± 2 |

**^*^** The Diabetes Knowledge Test’s summary score ranges from 0 (no questions correct) to 9 (all questions correct).

**^†^** The Diabetes Distress Scale score ranges from 17 (no distress) to 102 (severe distress).

**^‡^** The Interpersonal Processes of Care score ranges from 1 (poor relationship with healthcare provider) to 5 (good relationship with healthcare provider).

**^§^** The self monitoring of blood glucose value ranges from 0 (checked blood glucose on no days of the week as recommended by healthcare provider) to 7 (checked blood glucose on all 7 days of the week as recommended by healthcare provider).

**Supplementary Table 8:** Self-reported outcomes among patients, by country

|  | **All patients**  **N=4341** | **Japan**  **N=157** | **South Korea**  **N=141** | **Spain**  **N=148** | **Turkey**  **N=283** | **UAE**  **N=110** | **UK**  **N=126** |
| --- | --- | --- | --- | --- | --- | --- | --- |
|  | *mean ± SD* | | | | | | |
| **Diabetes Knowledge Test score^*^** | 5 ± 2 | 6 ± 2 | 3 ± 2 | 5 ± 2 | 4 ± 2 | 6 ± 2 | 5 ± 2 |
|  |  |  |  |  |  |  |  |
| **Diabetes Distress Scale score^†^** | 38 ± 19 | 31 ± 13 | 40 ± 17 | 36 ± 18 | 55 ± 21 | 51 ± 22 | 29 ± 13 |
|  |  |  |  |  |  |  |  |
| **Interpersonal Processes of Care score^‡^** | 3 ± 0.5 | 3 ± 0.5 | 3 ± 0.5 | 3 ± 0.5 | 3 ± 0.5 | 3 ± 0.5 | 3 ± 0.5 |
|  |  |  |  |  |  |  |  |
| **Self-monitoring of blood glucose^§^** | 3 ± 3 | 3 ± 3 | 4 ± 3 | 4 ± 3 | 3 ± 3 | 4 ± 2 | 5 ± 3 |

**^*^** The Diabetes Knowledge Test’s summary score ranges from 0 (no questions correct) to 9 (all questions correct).

**^†^** The Diabetes Distress Scale score ranges from 17 (no distress) to 102 (severe distress).

**^‡^** The Interpersonal Processes of Care score ranges from 1 (poor relationship with healthcare provider) to 5 (good relationship with healthcare provider).

**^§^** The self monitoring of blood glucose value ranges from 0 (checked blood glucose on no days of the week as recommended by healthcare provider) to 7 (checked blood glucose on all 7 days of the week as recommended by healthcare provider).

**MOSAIc baseline analyses: complete case approach**

**Supplementary Table 9:** Baseline demographic characteristics of 4,341 patients enrolled in the MOSAIc study, complete case analysis

|  | **All patients**  **N=4341** | **China**  **N=373** | **Germany**  **N=149** | **India**  **N=918** | **Mexico**  **N=206** | **Russia**  **N=240** | **Saudi Arabia**  **N=226** | **USA**  **N=540** |
| --- | --- | --- | --- | --- | --- | --- | --- | --- |
|  | *N (%) or mean ± SD* | | | | | | | |
| **Demographics** |  |  |  |  |  |  |  |  |
| Age, years | 61 ± 11 | 60 ± 10 | 70 ± 10 | 58 ± 10 | 60 ± 12 | 64 ± 10 | 57 ± 10 | 64 ± 12 |
| Female gender | 2176 (50) | 209 (56) | 69 (46) | 405 (44) | 117 (57) | 168 (70) | 135 (60) | 284 (53) |
| Education |  |  |  |  |  |  |  |  |
| <12 grade | 1797 (45) | 185 (50) | 105 (78) | 362 (41) | 120 (60) | 30 (13) | 88 (43) | 105 (22) |
| High school graduate or partial college | 1240 (31) | 131 (35) | 16 (12) | 192 (22) | 38 (19) | 89 (39) | 52 (25) | 253 (52) |
| > College degree | 969 (24) | 55 (15) | 14 (10) | 334 (38) | 42 (21) | 111 (48) | 67 (32) | 131 (27) |
| Marital status |  |  |  |  |  |  |  |  |
| Married or living with a significant other | 3253 (81) | 338 (92) | 101 (71) | 858 (94) | 149 (75) | 157 (69) | 175 (78) | 324 (66) |
| Single | 786 (19) | 31 (8) | 41 (29) | 58 (6) | 50 (25) | 69 (31) | 49 (22) | 168 (34) |
| Employment |  |  |  |  |  |  |  |  |
| Manual labor | 677 (22) | 57 (16) | 14 (11) | 128 (21) | 50 (37) | 15 (7) | 55 (32) | 48 (13) |
| Professional labor | 1052 (34) | 151 (43) | 20 (16) | 206 (33) | 45 (33) | 121 (57) | 72 (42) | 130 (36) |
| Skilled labor | 1335 (44) | 144 (41) | 89 (72) | 288 (46) | 40 (30) | 76 (36) | 46 (27) | 187 (51) |
| Insurance type |  |  |  |  |  |  |  |  |
| Private | 917 (23) | 38 (10) | 1 (1) | 289 (33) | 30 (16) | 6 (3) | 2 (1) | 296 (63) |
| Public | 2,229 (56) | 293 (80) | 136 (99) | 155 (17) | 106 (56) | 215 (92) | 152 (76) | 151 (32) |
| Uninsured | 848 (21) | 37 (10) | 0 (0) | 446 (50) | 52 (28) | 12 (5) | 47 (23) | 23 (5) |
| Alcohol consumption |  |  |  |  |  |  |  |  |
| Current | 575 (14) | 35 (10) | 31 (21) | 47 (5) | 28 (14) | 3 (1) | 1 (0) | 142 (27) |
| Past | 998 (24) | 92 (25) | 40 (27) | 93 (10) | 43 (22) | 15 (6) | 1 (0) | 237 (45) |
| Smoking status |  |  |  |  |  |  |  |  |
| Current | 565 (14) | 67 (18) | 16 (11) | 54 (6) | 31 (16) | 23 (10) | 14 (7) | 88 (17) |

**Supplementary Table 10:** Baseline clinical characteristics of 4,341 patients enrolled in MOSAIc study, as recorded in the patient medical record, complete case analysis

|  | **All patients**  **N=4341** | **China**  **N=373** | **Germany**  **N=149** | **India**  **N=918** | **Mexico**  **N=206** | **Russia**  **N=240** | **Saudi Arabia**  **N=226** | **USA**  **N=540** |
| --- | --- | --- | --- | --- | --- | --- | --- | --- |
|  | *N (%) or mean ± SD* | | | | | | | |
| Diabetes duration, in years | 12 ± 8 | 11 ± 8 | 14 ± 8 | 12 ± 8 | 13 ± 9 | 11 ± 7 | 11 ± 7 | 13 ± 8 |
| *Physician’s* HbA1c goal for the patient (%, mmHg) | 6.9 ± 0.6 | 6.6 ± 0.9 | 6.9 ± 0.5 | 6.9 ± 0.5 | 6.7 ± 0.7 | 6.7 ± 0.5 | 7.2 ± 0.3 | 6.8 ± 0.5 |
| *Physician’s* HbA1c goal for the patient (mmol/mol) | 52 ± 7 | 49 ± 10 | 52 ± 6 | 52 ± 6 | 50 ± 8 | 50 ± 6 | 55 ± 3 | 51 ± 6 |
|  |  |  |  |  |  |  |  |  |
| **Laboratory values** |  |  |  |  |  |  |  |  |
| HbA1c level (%, mmHg) | 8.1 ± 1.7 | 7.5 ± 1.8 | 7.5 ± 1.4 | 8.6 ± 1.7 | 8.9 ± 2.6 | 7.6 ± 1.2 | 9.1 ± 2.3 | 7.9 ± 1.5 |
| HbA1c level (mmol/mol) | 65 ± 19 | 58 ± 20 | 58 ± 15 | 70 ± 19 | 74 ± 28 | 60 ± 13 | 76 ± 25 | 63 ± 17 |
|  |  |  |  |  |  |  |  |  |
| **Biometric measurements** |  |  |  |  |  |  |  |  |
| Systolic blood pressure (mmHg) | 132.4 ± 16.2 | 130.1 ± 15.7 | 137.9 ± 17.1 | 131.3 ± 14.5 | 130.5 ± 17.6 | 136.3 ± 11.9 | 134.5 ± 13.3 | 132.0 ± 18.4 |
| Body mass index (kg/m^2^) | 30 ± 6 | 25 ± 3 | 30 ± 5 | 27 ± 5 | 28 ± 5 | 32 ± 5 | 32 ± 6 | 34 ± 8 |
|  |  |  |  |  |  |  |  |  |
| **Diabetes-related complications** |  |  |  |  |  |  |  |  |
| Amputation | 45 (1) | 0 (0) | 3 (2) | 4 (0) | 4 (2) | 3 (1) | 2 (1) | 10 (2) |
| Gastroparesis | 102 (3) | 11 (3) | 2 (1) | 7 (1) | 3 (2) | 18 (8) | 21 (10) | 11 (2) |
| Nephropathy | 685 (17) | 70 (19) | 37 (25) | 72 (8) | 17 (9) | 57 (25) | 49 (23) | 97 (19) |
| Neuropathy | 1194 (29) | 118 (32) | 56 (38) | 216 (25) | 49 (24) | 151 (64) | 76 (39) | 201 (39) |
| Retinopathy | 954 (24) | 84 (23) | 22 (15) | 135 (16) | 30 (15) | 111 (48) | 105 (53) | 72 (14) |
|  |  |  |  |  |  |  |  |  |
| **Diagnostic history of:** |  |  |  |  |  |  |  |  |
| Coronary artery disease | 770 (19) | 75 (20) | 28 (20) | 103 (11) | 3 (2) | 130 (55) | 58 (26) | 130 (26) |
| Congestive heart failure | 237 (6) | 21 (6) | 19 (13) | 19 (2) | 3 (2) | 81 (34) | 5 (2) | 31 (6) |
| Depression | 370 (9) | 13 (4) | 13 (9) | 22 (2) | 26 (13) | 15 (6) | 8 (4) | 115 (22) |
| Hypertension | 2994 (71) | 199 (53) | 133 (90) | 627 (69) | 113 (55) | 208 (87) | 143 (64) | 463 (86) |
| Hyperlipidemia | 2484 (60) | 204 (55) | 95 (66) | 381 (43) | 85 (43) | 139 (60) | 155 (69) | 442 (82) |
| Myocardial infarction | 271 (6) | 7 (2) | 10 (7) | 21 (2) | 5 (3) | 47 (20) | 18 (8) | 44 (8) |
| Stroke | 151 (4) | 18 (5) | 9 (7) | 11 (1) | 1 (1) | 14 (6) | 4 (2) | 31 (6) |

**Supplementary Table 11:** Characteristics of insulin and oral antidiabetic medication use among patients, by country

|  | **All patients**  **N=4341** | **China**  **N=373** | **Germany**  **N=149** | **India**  **N=918** | **Mexico**  **N=206** | **Russia**  **N=240** | **Saudi Arabia**  **N=226** | **USA**  **N=540** |
| --- | --- | --- | --- | --- | --- | --- | --- | --- |
|  | *N (%)* | | | | | | | |
| **Any insulin regimen together with:** |  |  |  |  |  |  |  |  |
| 0 oral antidiabetic drugs | 1449 (33) | 203 (54) | 90 (60) | 161 (18) | 120 (58) | 127 (53) | 56 (25) | 149 (28) |
| 1 oral antidiabetic drug | 1388 (32) | 109 (29) | 41 (28) | 243 (27) | 58 (28) | 69 (29) | 41 (18) | 224 (41) |
| 2 oral antidiabetic drugs | 977 (23) | 52 (14) | 14 (9) | 282 (31) | 21 (10) | 37 (15) | 78 (35) | 132 (24) |
| 3 or more oral antidiabetic drugs | 527 (12) | 9 (2) | 4 (3) | 232 (25) | 7 (3) | 7 (3) | 51 (23) | 35 (6) |
| **Insulin regimen** |  |  |  |  |  |  |  |  |
| Basal insulin only | 2234 (51) | 60 (16) | 65 (44) | 252 (27) | 146 (71) | 197 (82) | 140 (62) | 355 (66) |
| Basal + short-acting insulin only | 237 (5) | 3 (1) | 6 (4) | 11 (1) | 15 (7) | 13 (5) | 1 (0) | 32 (6) |
| Mixed insulin only | 1310 (30) | 249 (67) | 66 (44) | 522 (57) | 34 (17) | 19 (8) | 40 (18) | 93 (17) |
| Short-acting insulin only | 175 (4) | 13 (4) | 5 (3) | 43 (5) | 9 (4) | 10 (4) | 0 (0) | 20 (4) |
| Other insulin combinations | 385 (9) | 48 (13) | 7 (5) | 90 (10) | 2 (1) | 1 (0) | 45 (20) | 40 (7) |
| **Insulin injection frequency** |  |  |  |  |  |  |  |  |
| Once per day | 2579 (59) | 128 (34) | 80 (54) | 415 (45) | 152 (74) | 161 (67) | 147 (65) | 373 (69) |
| Twice per day | 1585 (37) | 220 (59) | 58 (39) | 494 (54) | 51 (25) | 77 (32) | 78 (35) | 144 (27) |
| Three times per day | 177 (4) | 25 (7) | 11 (7) | 9 (1) | 3 (2) | 2 (1) | 1 (0) | 23 (4) |
| **Insulin delivery device** |  |  |  |  |  |  |  |  |
| Pen | 2871 (74) | 358 (100) | 138 (95) | 493 (58) | 59 (32) | 211 (93) | 141 (63) | 208 (44) |
| Syringe | 1023 (26) | 1 (0) | 8 (6) | 354 (42) | 127 (68) | 16 (7) | 82 (37) | 262 (56) |

**Supplementary Table 12:** Self-reported outcomes among patients, by country

|  | **All patients**  **N=4341** | **China**  **N=373** | **Germany**  **N=149** | **India**  **N=918** | **Mexico**  **N=206** | **Russia**  **N=240** | **Saudi Arabia**  **N=226** | **USA**  **N=540** |
| --- | --- | --- | --- | --- | --- | --- | --- | --- |
|  | *mean ± SD* | | | | | | | |
| **Diabetes Knowledge Test score^*^** | 5 ± 2 | 7 ± 2 | 6 ± 2 | 4 ± 2 | 4 ± 2 | 6 ± 2 | 4 ± 2 | 5 ± 2 |
|  |  |  |  |  |  |  |  |  |
| **Diabetes Distress Scale score^†^** | 38 ± 19 | 28 ± 11 | 24 ± 8 | 38 ± 19 | 39 ± 21 | 49 ± 18 | 52 ± 17 | 33 ± 15 |
|  |  |  |  |  |  |  |  |  |
| **Interpersonal Processes of Care score^‡^** | 3 ± 0.5 | 3 ± 0.5 | 3 ± 0.4 | 3 ± 0.5 | 3 ± 0.6 | 3 ± 0.5 | 3 ± 0.5 | 3 ± 0.4 |
|  |  |  |  |  |  |  |  |  |
| **Self-monitoring of blood glucose^§^** | 3 ± 3 | 2 ± 2 | 5 ± 2 | 2 ± 2 | 3 ± 3 | 4 ± 2 | 2 ± 2 | 5 ± 3 |

**^*^** The Diabetes Knowledge Test’s summary score ranges from 0 (no questions correct) to 9 (all questions correct).

**^†^** The Diabetes Distress Scale score ranges from 17 (no distress) to 102 (severe distress).

**^‡^** The Interpersonal Processes of Care score ranges from 1 (poor relationship with healthcare provider) to 5 (good relationship with healthcare provider).

**^§^** The self monitoring of blood glucose value ranges from 0 (checked blood glucose on no days of the week as recommended by healthcare provider) to 7 (checked blood glucose on all 7 days of the week as recommended by healthcare provider).

**Supplementary Table 13:** Baseline demographic characteristics of 4,341 patients enrolled in the MOSAIc study, complete case analysis

|  | **All patients**  **N=4341** | **Argentina**  **N=160** | **Brazil**  **N=155** | **Canada**  **N=149** | **Israel**  **N=100** | **Italy**  **N=160** |
| --- | --- | --- | --- | --- | --- | --- |
|  | *N (%) or mean ± SD* | | | | | |
| **Demographics** |  |  |  |  |  |  |
| Age, years | 61 ± 11 | 65 ± 11 | 61 ± 10 | 66 ± 11 | 62 ± 9 | 64 ± 11 |
| Female gender | 2176 (50) | 76 (48) | 100 (65) | 63 (42) | 45 (45) | 60 (38) |
| Education |  |  |  |  |  |  |
| <12 grade | 1797 (45) | 95 (63) | 78 (58) | 64 (44) | 31 (32) | 93 (66) |
| High school graduate or partial college | 1240 (31) | 33 (22) | 28 (21) | 65 (45) | 47 (49) | 43 (30) |
| > College degree | 969 (24) | 24 (16) | 28 (21) | 15 (10) | 19 (20) | 6 (4) |
| Marital status |  |  |  |  |  |  |
| Married or living with a significant other | 3253 (81) | 109 (72) | 91 (62) | 101 (71) | 85 (87) | 118 (81) |
| Single | 786 (19) | 42 (28) | 56 (38) | 42 (29) | 13 (13) | 28 (19) |
| Employment |  |  |  |  |  |  |
| Manual labor | 677 (22) | 24 (30) | 40 (40) | 31 (29) | 11 (16) | 24 (23) |
| Professional labor | 1052 (34) | 21 (26) | 41 (41) | 30 (28) | 27 (40) | 21 (20) |
| Skilled labor | 1335 (44) | 36 (44) | 20 (20) | 46 (43) | 30 (44) | 59 (57) |
| Insurance type |  |  |  |  |  |  |
| Private | 917 (23) | 56 (36) | 48 (34) | 41 (31) | 15 (19) | 8 (6) |
| Public | 2,229 (56) | 65 (42) | 76 (54) | 67 (51) | 61 (75) | 49 (34) |
| Uninsured | 848 (21) | 35 (22) | 16 (11) | 24 (18) | 5 (6) | 89 (61) |
| Alcohol consumption |  |  |  |  |  |  |
| Current | 575 (14) | 29 (18) | 12 (9) | 41 (30) | 1 (1) | 30 (20) |
| Past | 998 (24) | 38 (24) | 25 (19) | 66 (52) | 1 (1) | 57 (37) |
| Smoking status |  |  |  |  |  |  |
| Current | 565 (14) | 17 (11) | 13 (9) | 19 (13) | 13 (13) | 33 (21) |

**Supplementary Table 14:** Baseline demographic characteristics of 4,341 patients enrolled in the MOSAIc study, complete case analysis

|  | **All patients**  **N=4341** | **Japan**  **N=157** | **South Korea**  **N=141** | **Spain**  **N=148** | **Turkey**  **N=283** | **UAE**  **N=110** | **UK**  **N=126** |
| --- | --- | --- | --- | --- | --- | --- | --- |
|  | *N (%) or mean ± SD* | | | | | | |
| **Demographics** |  |  |  |  |  |  |  |
| Age, years | 61 ± 11 | 64 ± 13 | 62 ± 11 | 68 ± 12 | 58 ± 9 | 55 ± 10 | 65 ± 11 |
| Female gender | 2176 (50) | 61 (39) | 51 (36) | 78 (53) | 142 (50) | 54 (49) | 59 (47) |
| Education |  |  |  |  |  |  |  |
| <12 grade | 1797 (45) | 29 (21) | 62 (47) | 91 (80) | 175 (73) | 36 (37) | 48 (52) |
| High school graduate or partial college | 1240 (31) | 85 (60) | 50 (38) | 18 (16) | 46 (19) | 25 (26) | 29 (32) |
| > College degree | 969 (24) | 27 (19) | 19 (15) | 5 (4) | 20 (8) | 37 (38) | 15 (16) |
| Marital status |  |  |  |  |  |  |  |
| Married or living with a significant other | 3253 (81) | 82 (76) | 122 (88) | 106 (75) | 146 (87) | 97 (88) | 94 (78) |
| Single | 786 (19) | 26 (24) | 17 (12) | 35 (25) | 22 (13) | 13 (12) | 26 (22) |
| Employment |  |  |  |  |  |  |  |
| Manual labor | 677 (22) | 14 (12) | 28 (35) | 42 (41) | 70 (41) | 8 (13) | 18 (19) |
| Professional labor | 1052 (34) | 34 (30) | 17 (21) | 12 (12) | 35 (21) | 41 (66) | 28 (30) |
| Skilled labor | 1335 (44) | 65 (58) | 35 (44) | 48 (47) | 64 (38) | 13 (21) | 49 (52) |
| Insurance type |  |  |  |  |  |  |  |
| Private | 917 (23) | 36 (26) | 12 (9) | 1 (1) | 2 (1) | 27 (30) | 9 (9) |
| Public | 2,229 (56) | 89 (65) | 108 (82) | 137 (98) | 244 (98) | 58 (64) | 67 (64) |
| Uninsured | 848 (21) | 12 (9) | 12 (9) | 2 (1) | 2 (1) | 6 (7) | 28 (27) |
| Alcohol consumption |  |  |  |  |  |  |  |
| Current | 575 (14) | 55 (39) | 34 (24) | 20 (14) | 6 (3) | 5 (5) | 55 (50) |
| Past | 998 (24) | 91 (65) | 59 (42) | 41 (28) | 12 (6) | 7 (7) | 80 (70) |
| Smoking status |  |  |  |  |  |  |  |
| Current | 565 (14) | 38 (26) | 25 (19) | 21 (15) | 59 (22) | 15 (16) | 19 (16) |

**Supplementary Table 15:** Baseline clinical characteristics of 4,341 patients enrolled in MOSAIc study, as recorded in the patient medical record

|  | **All patients**  **N=4341** | **Argentina**  **N=160** | **Brazil**  **N=155** | **Canada**  **N=149** | **Israel**  **N=100** | **Italy**  **N=160** |
| --- | --- | --- | --- | --- | --- | --- |
|  | *N (%) or mean ± SD* | | | | | |
| Diabetes duration, in years | 12 ± 8 | 13 ± 10 | 13 ± 8 | 15 ± 8 | 14 ± 8 | 13 ± 8 |
| *Physician’s* HbA1c goal for the patient (%, mmHg) | 6.9 ± 0.6 | 7.0 ± 0.5 | 7.0 ± 0.3 | 6.9 ± 0.5 | 7.2 ± 0.6 | 6.7 ± 0.6 |
| *Physician’s* HbA1c goal for the patient (mmol/mol) | 52 ± 7 | 53 ± 6 | 53 ± 3 | 52 ± 6 | 55 ± 7 | 50 ± 7 |
|  |  |  |  |  |  |  |
| **Laboratory values** |  |  |  |  |  |  |
| HbA1c level (%, mmHg) | 8.1 ± 1.7 | 8.1 ± 1.8 | 8.3 ± 1.9 | 8.0 ± 1.2 | 7.7 ± 1.2 | 8.0 ± 1.3 |
| HbA1c level (mmol/mol) | 65 ± 19 | 65 ± 20 | 67 ± 21 | 64 ± 13 | 61 ± 13 | 64 ± 14 |
|  |  |  |  |  |  |  |
| **Biometric measurements** |  |  |  |  |  |  |
| Systolic blood pressure (mmHg) | 132.4 ± 16.2 | 133.8 ± 13.0 | 135.5 ± 18.8 | 132.0 ± 16.5 | 129.6 ± 15.2 | 136.1 ± 18.5 |
| Body mass index (kg/m^2^) | 30 ± 6 | 31 ± 6 | 30 ± 5 | 32 ± 7 | 30 ± 6 | 29 ± 5 |
|  |  |  |  |  |  |  |
| **Diabetes-related complications** |  |  |  |  |  |  |
| Amputation | 45 (1) | 4 (3) | 6 (4) | 0 (0) | 2 (2) | 1 (1) |
| Gastroparesis | 102 (3) | 3 (2) | 2 (2) | 4 (3) | 0 (0) | 1 (1) |
| Nephropathy | 685 (17) | 14 (9) | 17 (12) | 27 (19) | 26 (26) | 23 (15) |
| Neuropathy | 1194 (29) | 14 (9) | 22 (15) | 36 (25) | 18 (18) | 34 (23) |
| Retinopathy | 954 (24) | 24 (15) | 24 (17) | 30 (21) | 27 (27) | 52 (33) |
|  |  |  |  |  |  |  |
| **Diagnostic history of:** |  |  |  |  |  |  |
| Coronary artery disease | 770 (19) | 14 (9) | 12 (9) | 37 (25) | 28 (28) | 28 (18) |
| Congestive heart failure | 237 (6) | 0 (0) | 3 (2) | 4 (3) | 8 (8) | 4 (3) |
| Depression | 370 (9) | 4 (3) | 16 (14) | 24 (16) | 7 (7) | 9 (6) |
| Hypertension | 2994 (71) | 112 (70) | 110 (73) | 125 (85) | 76 (76) | 113 (71) |
| Hyperlipidemia | 2484 (60) | 81 (51) | 93 (62) | 112 (76) | 86 (87) | 97 (61) |
| Myocardial infarction | 271 (6) | 12 (8) | 6 (4) | 18 (12) | 19 (19) | 15 (10) |
| Stroke | 151 (4) | 4 (3) | 5 (4) | 6 (4) | 10 (10) | 8 (5) |

**Supplementary Table 16:** Baseline clinical characteristics of 4,341 patients enrolled in MOSAIc study, as recorded in the patient medical record

|  | **All patients**  **N=4341** | **Japan**  **N=157** | **South Korea**  **N=141** | **Spain**  **N=148** | **Turkey**  **N=283** | **UAE**  **N=110** | **UK**  **N=126** |
| --- | --- | --- | --- | --- | --- | --- | --- |
|  | *N (%) or mean ± SD* | | | | | | |
| Diabetes duration, in years | 12 ± 8 | 14 ± 9 | 15 ± 9 | 13 ± 8 | 11 ± 7 | 15 ± 8 | 12 ± 6 |
| *Physician’s* HbA1c goal for the patient (%, mmHg) | 6.9 ± 0.6 | 6.3 ± 0.6 | 7.1 ± 0.5 | 7.0 ± 1.0 | 6.8 ± 0.6 | 6.9 ± 0.2 | 7.4 ± 0.4 |
| *Physician’s* HbA1c goal for the patient (mmol/mol) | 52 ± 7 | 45 ± 7 | 54 ± 6 | 53 ± 11 | 51 ± 7 | 52 ± 2 | 57 ± 4 |
|  |  |  |  |  |  |  |  |
| **Laboratory values** |  |  |  |  |  |  |  |
| HbA1c level (%, mmHg) | 8.1 ± 1.7 | 7.6 ± 1.3 | 8.1 ± 1.2 | 8.0 ± 1.5 | 8.9 ± 2.1 | 8.2 ± 1.7 | 8.3 ± 1.5 |
| HbA1c level (mmol/mol) | 65 ± 19 | 60 ± 14 | 65 ± 13 | 64 ± 16 | 74 ± 23 | 66 ± 19 | 67 ± 16 |
|  |  |  |  |  |  |  |  |
| **Biometric measurements** |  |  |  |  |  |  |  |
| Systolic blood pressure (mmHg) | 132.4 ± 16.2 | 131.2 ± 15.5 | 126.6 ± 15.5 | 134.9 ± 16.5 | 131.4 ± 17.9 | 131.4 ± 17.4 | 136.9 ± 19.8 |
| Body mass index (kg/m^2^) | 30 ± 6 | 25 ± 4 | 26 ± 4 | 30 ± 5 | 31 ± 5 | 32 ± 7 | 33 ± 6 |
|  |  |  |  |  |  |  |  |
| **Diabetes-related complications** |  |  |  |  |  |  |  |
| Amputation | 45 (1) | 0 (0) | 1 (1) | 1 (1) | 2 (1) | 0 (0) | 2 (2) |
| Gastroparesis | 102 (3) | 5 (3) | 0 (0) | 9 (6) | 2 (1) | 1 (1) | 2 (2) |
| Nephropathy | 685 (17) | 43 (28) | 50 (36) | 29 (20) | 20 (11) | 23 (22) | 14 (13) |
| Neuropathy | 1194 (29) | 24 (16) | 56 (42) | 17 (12) | 45 (25) | 40 (39) | 21 (18) |
| Retinopathy | 954 (24) | 40 (26) | 69 (50) | 28 (19) | 25 (15) | 31 (31) | 45 (39) |
|  |  |  |  |  |  |  |  |
| **Diagnostic history of:** |  |  |  |  |  |  |  |
| Coronary artery disease | 770 (19) | 13 (8) | 22 (16) | 23 (16) | 24 (13) | 17 (17) | 25 (21) |
| Congestive heart failure | 237 (6) | 4 (3) | 1 (1) | 25 (17) | 0 (0) | 1 (1) | 8 (7) |
| Depression | 370 (9) | 5 (3) | 12 (9) | 25 (17) | 13 (7) | 12 (12) | 31 (27) |
| Hypertension | 2994 (71) | 86 (56) | 96 (69) | 112 (76) | 92 (43) | 89 (82) | 97 (82) |
| Hyperlipidemia | 2484 (60) | 94 (61) | 88 (64) | 97 (66) | 69 (36) | 98 (91) | 68 (62) |
| Myocardial infarction | 271 (6) | 13 (8) | 9 (7) | 9 (6) | 5 (3) | 4 (4) | 9 (8) |
| Stroke | 151 (4) | 5 (3) | 9 (7) | 5 (4) | 1 (1) | 5 (5) | 5 (4) |

**Supplementary Table 17:** Characteristics of insulin and oral antidiabetic medication use among patients, by country

|  | **All patients**  **N=4,341** | **Argentina**  **N=160** | **Brazil**  **N=155** | **Canada**  **N=149** | **Israel**  **N=100** | **Italy**  **N=160** |
| --- | --- | --- | --- | --- | --- | --- |
|  | *N (%)* | | | | | |
| **Any insulin regimen together with:** |  |  |  |  |  |  |
| 0 oral antidiabetic drugs | 1449 (33) | 72 (45) | 32 (21) | 55 (37) | 25 (25) | 33 (21) |
| 1 oral antidiabetic drug | 1388 (32) | 61 (38) | 59 (38) | 42 (28) | 41 (41) | 46 (29) |
| 2 oral antidiabetic drugs | 977 (23) | 25 (16) | 55 (36) | 31 (21) | 23 (23) | 41 (26) |
| 3 or more oral antidiabetic drugs | 527 (12) | 2 (1) | 9 (6) | 21 (14) | 11 (11) | 40 (25) |
| **Insulin regimen** |  |  |  |  |  |  |
| Basal insulin only | 2234 (51) | 103 (64) | 122 (79) | 92 (62) | 65 (65) | 118 (74) |
| Basal + short-acting insulin only | 237 (5) | 9 (6) | 30 (19) | 8 (5) | 16 (16) | 17 (11) |
| Mixed insulin only | 1310 (30) | 36 (23) | 0 (0) | 24 (16) | 6 (6) | 3 (2) |
| Short-acting insulin only | 175 (4) | 1 (1) | 1 (1) | 0 (0) | 0 (0) | 6 (4) |
| Other insulin combinations | 385 (9) | 11 (7) | 2 (1) | 25 (17) | 13 (13) | 16 (10) |
| **Insulin injection frequency** |  |  |  |  |  |  |
| Once per day | 2579 (59) | 71 (44) | 79 (51) | 84 (56) | 73 (73) | 123 (77) |
| Twice per day | 1585 (37) | 68 (43) | 56 (36) | 55 (37) | 20 (20) | 20 (13) |
| Three times per day | 177 (4) | 21 (13) | 20 (13) | 10 (7) | 7 (7) | 17 (11) |
| **Insulin delivery device** |  |  |  |  |  |  |
| Pen | 2871 (74) | 129 (99) | 24 (16) | 140 (97) | 65 (81) | 95 (85) |
| Syringe | 1023 (26) | 1 (1) | 122 (84) | 5 (3) | 15 (19) | 17 (15) |

**Supplementary Table 18:** Characteristics of insulin and oral antidiabetic medication use among patients, by country

|  | **All patients**  **N=4341** | **Japan**  **N=157** | **South Korea**  **N=141** | **Spain**  **N=148** | **Turkey**  **N=283** | **UAE**  **N=110** | **UK**  **N=126** |
| --- | --- | --- | --- | --- | --- | --- | --- |
|  | *N (%)* | | | | | | |
| **Any insulin regimen together with:** |  |  |  |  |  |  |  |
| 0 oral antidiabetic drugs | 1449 (33) | 29 (19) | 50 (36) | 39 (26) | 168 (59) | 13 (12) | 27 (21) |
| 1 oral antidiabetic drug | 1388 (32) | 35 (22) | 70 (50) | 75 (51) | 82 (29) | 25 (23) | 67 (53) |
| 2 oral antidiabetic drugs | 977 (23) | 34 (22) | 19 (14) | 27 (18) | 31 (11) | 48 (44) | 27 (21) |
| 3 or more oral antidiabetic drugs | 527 (12) | 59 (38) | 2 (1) | 7 (5) | 2 (1) | 24 (22) | 5 (4) |
| **Insulin regimen** |  |  |  |  |  |  |  |
| Basal insulin only | 2234 (51) | 110 (70) | 81 (57) | 106 (72) | 85 (30) | 66 (60) | 71 (56) |
| Basal + short-acting insulin only | 237 (5) | 11 (7) | 12 (9) | 18 (12) | 11 (4) | 23 (21) | 1 (1) |
| Mixed insulin only | 1310 (30) | 17 (11) | 14 (10) | 9 (6) | 123 (43) | 10 (9) | 45 (36) |
| Short-acting insulin only | 175 (4) | 13 (8) | 4 (3) | 9 (6) | 36 (13) | 1 (1) | 4 (3) |
| Other insulin combinations | 385 (9) | 6 (4) | 30 (21) | 6 (4) | 28 (10) | 10 (9) | 5 (4) |
| **Insulin injection frequency** |  |  |  |  |  |  |  |
| Once per day | 2579 (59) | 114 (73) | 107 (76) | 91 (62) | 208 (74) | 80 (73) | 93 (74) |
| Twice per day | 1585 (37) | 37 (24) | 33 (23) | 54 (37) | 71 (25) | 20 (18) | 29 (23) |
| Three times per day | 177 (4) | 6 (4) | 1 (1) | 3 (2) | 4 (1) | 10 (9) | 4 (3) |
| **Insulin delivery device** |  |  |  |  |  |  |  |
| Pen | 2871 (74) | 154 (100) | 125 (95) | 141 (99) | 208 (100) | 98 (98) | 84 (98) |
| Syringe | 1023 (26) | 0 (0) | 7 (5) | 1 (1) | 1 (1) | 2 (2) | 2 (2) |

**Supplementary Table 19:** Self-reported outcomes among patients, by country

|  | **All patients**  **N=4341** | **Argentina**  **N=160** | **Brazil**  **N=155** | **Canada**  **N=149** | **Israel**  **N=100** | **Italy**  **N=160** |
| --- | --- | --- | --- | --- | --- | --- |
|  | *mean ± SD* | | | | | |
| **Diabetes Knowledge Test score^*^** | 5 ± 2 | 5 ± 2 | 4 ± 2 | 5 ± 2 | 5 ± 2 | 6 ± 2 |
|  |  |  |  |  |  |  |
| **Diabetes Distress Scale score^†^** | 38 ± 19 | 37 ± 20 | 55 ± 23 | 27 ± 12 | 42 ± 19 | 37 ± 17 |
|  |  |  |  |  |  |  |
| **Interpersonal Processes of Care score^‡^** | 3 ± 0.5 | 3 ± 0.4 | 3 ± 0.5 | 3 ± 0.4 | 3 ± 0.5 | 3 ± 0.4 |
|  |  |  |  |  |  |  |
| **Self-monitoring of blood glucose^§^** | 3 ± 3 | 5 ± 3 | 3 ± 3 | 6 ± 2 | 5 ± 3 | 5 ± 2 |

**^*^** The Diabetes Knowledge Test’s summary score ranges from 0 (no questions correct) to 9 (all questions correct).

**^†^** The Diabetes Distress Scale score ranges from 17 (no distress) to 102 (severe distress).

**^‡^** The Interpersonal Processes of Care score ranges from 1 (poor relationship with healthcare provider) to 5 (good relationship with healthcare provider).

**^§^** The self monitoring of blood glucose value ranges from 0 (checked blood glucose on no days of the week as recommended by healthcare provider) to 7 (checked blood glucose on all 7 days of the week as recommended by healthcare provider).

**Supplementary Table 20:** Self-reported outcomes among patients, by country

|  | **All patients**  **N=4341** | **Japan**  **N=157** | **South Korea**  **N=141** | **Spain**  **N=148** | **Turkey**  **N=283** | **UAE**  **N=110** | **UK**  **N=126** |
| --- | --- | --- | --- | --- | --- | --- | --- |
|  | *mean ± SD* | | | | | | |
| **Diabetes Knowledge Test score^*^** | 5 ± 2 | 6 ± 2 | 3 ± 2 | 5 ± 2 | 4 ± 2 | 6 ± 2 | 5 ± 2 |
|  |  |  |  |  |  |  |  |
| **Diabetes Distress Scale score^†^** | 38 ± 19 | 31 ± 13 | 41 ± 15 | 37 ± 18 | 56 ± 21 | 52 ± 23 | 29 ± 13 |
|  |  |  |  |  |  |  |  |
| **Interpersonal Processes of Care score^‡^** | 3 ± 0.5 | 3 ± 0.5 | 3 ± 0.5 | 3 ± 0.5 | 3 ± 0.5 | 3 ± 0.5 | 3 ± 0.5 |
|  |  |  |  |  |  |  |  |
| **Self-monitoring of blood glucose^§^** | 3 ± 3 | 4 ± 3 | 4 ± 3 | 4 ± 3 | 3 ± 3 | 4 ± 2 | 5 ± 3 |

**^*^** The Diabetes Knowledge Test’s summary score ranges from 0 (no questions correct) to 9 (all questions correct).

**^†^** The Diabetes Distress Scale score ranges from 17 (no distress) to 102 (severe distress).

**^‡^** The Interpersonal Processes of Care score ranges from 1 (poor relationship with healthcare provider) to 5 (good relationship with healthcare provider).

**^§^** The self monitoring of blood glucose value ranges from 0 (checked blood glucose on no days of the week as recommended by healthcare provider) to 7 (checked blood glucose on all 7 days of the week as recommended by healthcare provider.
